# Real-world experience with long-term albumin in patients with cirrhosis and ascites

**DOI:** 10.1016/j.jhepr.2024.101221

**Published:** 2024-09-17

**Authors:** Enrico Pompili, Giacomo Zaccherini, Salvatore Piano, Pierluigi Toniutto, Antonio Lombardo, Stefania Gioia, Giulia Iannone, Clara De Venuto, Marta Tonon, Roberta Gagliardi, Maurizio Baldassarre, Greta Tedesco, Giorgio Bedogni, Marco Domenicali, Vito Di Marco, Silvia Nardelli, Vincenza Calvaruso, Davide Bitetto, Paolo Angeli, Paolo Caraceni

**Affiliations:** 1 Department of Medical and Surgical Sciences, Alma Mater Studiorum University of Bologna, Italy; 2 Unit of Semeiotics, Liver and Alcohol-related diseases, IRCCS Azienda Ospedaliero-Universitaria di Bologna, Italy; 3 Unit of Internal Medicine and Hepatology, Department of Medicine - DIMED, University and Hospital of Padova, Italy; 4 Hepatology and Liver Transplantation Unit, University Academic Hospital, Udine, Italy; 5 UOC di Gastroenterologia, Dipartimento di Promozione della Salute, Materno Infantile, Medicina Interna e Specialistica (PROMISE), University of Palermo, Italy; 6 Department of Translational and Precision Medicine, Sapienza University of Rome, Italy; 7 Department of Primary Health Care, Internal Medicine Unit Addressed to Frailty and Aging, "S. Maria Delle Croci" Hospital, AUSL Romagna, Ravenna, Italy

**Keywords:** decompensated cirrhosis, ascites, portal hypertension, human albumin, TIPS, paracentesis, refractory ascites, liver cirrhosis, outpatient, ANSWER

## Abstract

**Background & Aims:**

Long-term albumin (LTA) is currently standard of care for patients with decompensated cirrhosis in many Italian hepatology centres. In this real-life study, we aimed to describe patient, logistical and treatment-related characteristics in daily clinical practice and to identify predictors of response.

**Methods:**

We performed a multicentre, retrospective, observational study in patients with cirrhosis and ascites receiving LTA between 01/2016 and 02/2022 and followed until death, TIPS (transjugular intrahepatic portosystemic shunt) placement, transplantation or 02/2023.

**Results:**

A total of 312 patients, the majority with alcohol-related cirrhosis, were included. At baseline, median Child-Pugh, MELD, and MELD-Na were 8, 15, and 18, respectively. Ascites was grade 2 in 55% of patients, grade 3 in 35% and refractory in 27%, while 47% had received large volume paracentesis in the previous 6 months. Median LTA was 10 months with a median dose of 40 g/week. Ascites resolved to grade 0-1 in 34% of patients within the first 3 months and 56% by the end of treatment. Predictors of ascites resolution were age (*p =* 0.007), baseline grade of ascites (*p =* 0.007), no paracentesis in the previous 6 months (*p =* 0.001), aetiological treatment in the past 12 months or during LTA (*p =* 0.005), weekly albumin dose (*p =* 0.014) and serum albumin concentration of 40 g/L after 1 month of treatment (*p =* 0.017). Of the 83 patients with refractory ascites at inclusion, 26% had grade 0/1 ascites at the last observation. No severe albumin-related side-effects were reported and only 1% discontinued for logistical reasons.

**Conclusions:**

LTA is feasible as an outpatient treatment for the management of ascites. In the current study, ascites resolved in more than half of patients receiving LTA on top of diuretics, including in some with refractory ascites. Predictors of response to LTA provide useful information for tailoring treatment.

**Impact and implications:**

The ANSWER randomised-controlled trial has shown that long-term albumin treatment (LTA) is an effective approach for the management of patients with cirrhosis and ascites. This observational study provides novel information on target patients, modalities and length of treatment, predictors of ascites resolution, stopping criteria, and clinical trajectories of patients on treatment. LTA is a feasible option in the daily clinical practice for the management of ascites when given on top of diuretics. Rather than an alternative therapy, LTA should be integrated with the other treatment options already available for patients with difficult-to-treat ascites. The predictive factors of response identified in the present study can help physicians to individualise LTA and optimise the decision-making process.

## Highlights

• Patients with uncomplicated ascites are better candidates for long-term albumin.
• 34% of patients resolved ascites within 3 months and 56% at the end of treatment.
• Median length of treatment was 10 months and 24% of patients discontinued albumin due to improvement.
• Predictors of ascites response can help physicians to optimize the decision-making process.
• Only 1% of patients discontinued treatment due to logistical issues and no side effects were reported.

## Graphical abstract

**Figure:**
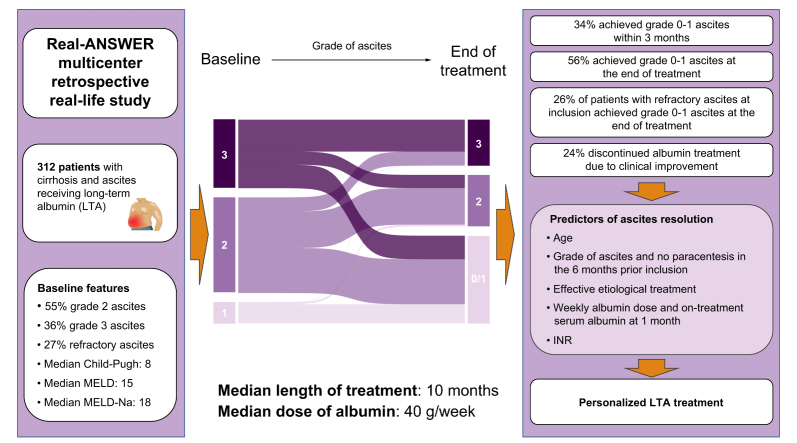

## Introduction

The ANSWER randomised-controlled trial (RCT) has shown that long-term albumin (LTA) treatment in patients with cirrhosis and uncomplicated ascites improves 18-month overall survival rates, facilitates the management of ascites, and lowers the incidence of severe complications, thus reducing hospitalizations and improving patients’ quality of life.^1^

Because of these results, LTA has been included among the medical options for the treatment of ascites by the Italian Association for the Study of the Liver (AISF) in the latest published guidelines^2^ and it has become standard of care in many hepatological centres throughout Italy, with the associated cost reimbursed by the National Healthcare System.

LTA is rarely used outside of Italy due to a reasonable scientific scepticism, as many physicians are waiting for confirmatory RCTs after the negative results of the MACHT trial^3^ and believe that the benefit of LTA can be also attributed to the more frequent interaction with healthcare professionals due to the weekly albumin infusions. Moreover, economic/logistical issues can limit the implementation of this treatment approach in many countries. Nevertheless, a recent international position statement by 33 hepatologists from 19 different countries has suggested that LTA can be considered for treating non-complicated ascites in a setting where budget and logistical issues can be resolved.^4^

The widespread use of LTA in Italy provides an opportunity to conduct large real-life studies to address questions left open by the ANSWER trial or that cannot be addressed by the rigid methodology of RCTs.

We therefore performed a retrospective observational real-life study in five hepatology centres, including patients with cirrhosis and ascites receiving LTA, with the specific aims of better defining: 1) the target population; 2) the modalities, feasibility and safety of treatment in daily clinical practice; 3) the response of ascites to LTA and the clinical and treatment-related factors predicting response; and 4) the clinical trajectories of patients receiving LTA.

## Patients and methods

### Study design

The Real-ANSWER is a multicentre, retrospective, observational study in patients with cirrhosis and ascites treated with LTA. The study was conducted in five Italian tertiary hepatological centres: the IRCCS Azienda Ospedaliero-Universitaria di Bologna, the University Hospital of Padua, the University Hospital of Palermo, the University Hospital of Udine and the University Hospital of Latina. Consecutive patients starting LTA from January 2016 to February 2022 and followed until death, liver transplantation (LT), transjugular intrahepatic portosystemic shunt (TIPS) placement or until February 28^th^, 2023, were included. Anthropometric, medical history, and clinical data were collected at baseline, while clinical data were regularly recorded during LTA treatment. After treatment interruption, data were also collected at the last available visit before death, LT, TIPS or February 28^th^, 2023.

Besides LTA, patients received human albumin (HA) during the study period for the established indications according to EASL guidelines (after large-volume paracentesis, treatment of acute kidney injury and hepatorenal syndrome [HRS] and spontaneous bacterial peritonitis [SBP]).^5^ The study protocol conforms to the ethical guidelines of the 1975 Declaration of Helsinki and has been approved by the local ethical committees at the coordinator centre (CE-AVEC) and at each participating centre. Due to the retrospective nature of the study, written informed consent was obtained from all patients who were alive and contactable, and waived for those who were deceased/unreachable.

### Inclusion and exclusion criteria

Inclusion criteria were: i) patients with diagnosis of cirrhosis based on clinical, laboratory, radiological and/or histological criteria; ii) prescription of regular HA infusions for at least 1 month; iii) age greater than 18 years. Exclusion criteria were: i) ongoing acute complications of cirrhosis (HRS, SBP and/or non-SBP-related bacterial infections, grade III/IV hepatic encephalopathy [HE], gastrointestinal bleeding); ii) ongoing acute-on-chronic liver failure defined according to the EASL-CLIF criteria;^5^ iii) hepatic surgery in the previous 14 days; iv) hepatocellular carcinoma (HCC) beyond Milan criteria or other extrahepatic malignancy; v) previous LT or other solid organ transplantation; vi) previous TIPS; vii) severe extrahepatic disease that, according to clinical judgement, may be the predominant factor affecting the patient's prognosis.

### Data collection and definitions

At the time of enrolment, demographic and anthropometric characteristics, medical history (aetiology, previous complications of cirrhosis, comorbidities) and clinical features (grade of ascites, concomitant decompensating events, oesophageal varices, HCC, laboratory data, prognostic scores) were collected.

The following data on LTA treatment were recorded: setting of administration, dosage, frequency and length of administration, changes of dosage, patient compliance/adherence, and indication for stopping treatment.

During treatment, clinical and laboratory data were recorded at 1, 3, 6, 9, 12, 18, 24 and 30 months at regular clinic visits when available and at the time of discontinuation of LTA. Ascites was graded and refractory ascites defined according to the International Club of Ascites (ICA) criteria.^6^ Ascites was assessed at baseline and at each time point by clinical examination. Resolution of ascites was defined as the achievement of grade 0-1 ascites in patients with grade 2-3 ascites at enrolment, *i.e*. ascites that was previously clinically detectable. Cases in which ascites was clinically absent (grade 0-1) at a single visit but present (grade 2 or 3) at the next visit were not considered "resolved". Patients underwent a complete abdominal ultrasound every 6 months as standard of care (surveillance for HCC). More frequent ultrasound or point-of-care ultrasound was performed at clinic visits when deemed appropriate, as well as further investigations such as CT or MRI. In patients with grade 1 ascites at enrolment (detectable only by ultrasound), resolution of ascites was defined as the absence of ascites (grade 0) on a follow-up ultrasound (or CT or MRI). Patients who received TIPS were included in the group that did not resolve ascites, while patients who died, received LT or were lost to follow-up were assessed based on the data reported at the last available visits. Overt HE was defined according to EASL guidelines.^7^

### Statistical analysis

For all continuous parameters the normality of distribution and homogeneity of variance were evaluated by the Shapiro Wilk and Levene tests, then variables were reported as mean (SD) or median (IQR) as appropriate. Accordingly, comparisons between groups were performed using the Student’s *t* test or the Mann-Whitney *U* test when appropriate. Categorical parameters were reported as frequency and percentage and compared using the Chi square or Fisher exact test. The cumulative incidence of treatment interruption due to clinical improvement and cumulative incidence of death during follow-up was estimated according to the Kaplan-Meier method.

Predictors of ascites resolution were identified by means of multivariable logistic regression analysis performed after multiple imputation of missing values by means of chained equations (MICE) with fully conditional specification (100 replicates) under a missing-at-random assumption (further details are reported in the supplementary appendix). For each predictor of the multivariable model the odds ratio (OR) and the 95% CI was reported.

All tests were two sided and *p* values less than 0.05 were considered statistically significant. Statistical analysis was performed using STATA (StataCorp LLC, College Station, TX, USA) version 18.

## Results

### Baseline characteristics and medical history

A total of 326 patients with cirrhosis and ascites received LTA during the study period. Fourteen patients were excluded due to incomplete data, and 312 patients were included in the final analyses. The distribution of patients among the centres was: 116 (36%) in Bologna, 89 (28%) in Padova, 44 (14%) in Udine, 39 (13%) in Palermo, and 24 (8%) in Latina.

The baseline characteristics at enrolment are detailed in Table 1. The median age was 63 years, with a predominance of males (70%), while the most common aetiology of cirrhosis was alcohol-related (60%) followed by metabolic dysfunction-associated steatotic liver disease (28%). Forty-seven (15%) patients were still active drinkers at enrolment. Fifty-six (18%) patients reached alcohol abstinence in the 6 months prior to inclusion in the study, while 17 patients (5%) became abstinent during follow-up. Thirty-eight (12%) patients had social difficulties, including 8 who were homeless and 19 with family problems.

**Table 1:** Baseline characteristics of patients with cirrhosis and ascites included in the study.

	N = 312
**Demographic data**	
Age (years)	63 (55–70)
Male sex, n (%)	219 (70)

**Aetiology of cirrhosis, n (%)**	
Alcohol	116 (37)
MASLD	48 (15)
Viral	45 (14)
Alcohol+viral	32 (10)
Alcohol+MASLD	40 (13)
Other	31 (10)

**Ascites, n (%)**	
Ascites grade 1	30 (10)
Ascites grade 2	171 (55)
Ascites grade 3	111 (35)
Refractory ascites	83 (27)
Previous paracentesis within 6 months prior to enrolment	147 (47)
1-3 paracenteses in last 6 months	83 (27)
≥4 paracenteses in last 6 months	64 (20)

**Medical history, n (%)**	
Presence of oesophageal varices	235 (75)
Low-risk varices	168 (54)
Moderate/high-risk varices	66 (21)
Previous overt HE	93 (30)
Previous gastrointestinal bleeding	60 (19)
Previous spontaneous bacterial peritonitis	30 (10)
Previous hepato-renal syndrome	22 (7)
HCC within Milan criteria	32 (10)
Active list for LT	41 (13)
Active alcohol consumption	47 (15)
Etiological treatment (<12 month or during the treatment)	114 (37)

**Concomitant medications**	
Antialdosteronic drugs (mg/day)	200 (100–200)
Furosemide (mg/day)	50 (25–75)
NSBB, n (%)	152 (49)
Fluoroquinolones for SBP prophylaxis, n (%)	11 (4)
Rifaximin for HE prophylaxis, n (%)	122 (39)

**Comorbidities**, n (%)	
Chronic heart disease	54 (17)
Chronic kidney disease	45 (14)
Chronic lung disease	30 (10)
Neurologic diseases	12 (4)
Diabetes	110 (35)

**Laboratory and hemodynamic data at inclusion**	
Hb (g/dl)	10.7 (9.5–12.3)
WBC (10^9^/L)	5.3 (3.9–7.1)
Platelets (10^9^/L)	102 (68–143)
Sodium (mmol/L)	136 (133–139)
Bilirubin (mg/dl)	2.1 (1.2–3.9)
Creatinine (mg/dl)	0.9 (0.8–1.2)
Albumin (g/L)	31 (27–35)
INR	1.4 (1.2–1.6)
MAP (mmHg)	87 (77–92)
HR (bpm)	74 (68–82)

**Prognostic scores**	
Child-Pugh score	8 (7–10)
MELD score	15 (11–18)
MELD-Na score	18 (15–21)

HCC, hepatocellular carcinoma; HE, hepatic encephalopathy; Hb, haemoglobin; HR, heart rate; INR, international normalised ratio; LT, liver transplantation; MASLD, metabolic dysfunction-associated steatotic liver disease; MAP, mean arterial pressure; MELD, model for end-stage liver disease; MELD-Na, model for end-stage liver disease incorporating serum sodium; NSBB, non-selective beta blockers; WBC, white blood cells.

Data are reported by median and interquartile range or absolute frequency and percentage (%) as appropriate.

At enrolment, ascites was grade 1 in 10% of patients, grade 2 in 55% and grade 3 in 35%, while 83 (27%) patients met the ICA criteria for the diagnosis of refractory ascites. At least one large volume paracentesis (LVP) was performed in 147 (47%) individuals in the 6 months prior to starting LTA: 83 (27%) patients received up to 3 paracenteses and 64 (20%) 4 or more paracenteses.

Apart from ascites, 96 (31%) patients presented previous episodes of other decompensating events: 30% overt HE and 19% gastrointestinal bleeding related to portal hypertension. Furthermore, 7% had a previous diagnosis of HRS and 10% of SBP. HCC was present in 10% of patients.

The median baseline albumin concentration was 31 g/L (27-35). The median Child Pugh score was 8 (7-10), median MELD was 15 (11-18), and median MELD-Na was 18 (15-21).

More than half of patients presents extrahepatic comorbidities, with the most prevalent being type 2 diabetes in 35% of the study population.

Finally, 95% of patients were receiving diuretics at the time of enrolment. The median dose of an antialdosteronic drug was 200 mg per day (100-200 mg), while that of furosemide was 50 mg per day (25-75 mg). Half of patients were taking non-selective beta-blockers and 39% rifaximin.

### Study follow-up

The median length of the whole follow-up until February 28^th^ 2023 was 16 months (IQR 9–31). Patient outcomes are reported in Fig. S1. The cumulative incidence of death during the whole follow-up period was 11% [95% CI 8–15] at 6 months, 23% [95% CI 18–28] at 12 months year and 35% [95% CI 30–42] at 18 months.

### Features of long-term albumin treatment

LTA treatment was initiated during hospitalisation and continued after discharge in 32% of cases, while in 68% of cases it was started in the outpatient clinic. The setting of albumin infusion was the outpatient clinic of the referral hospital in 56% of cases, and the territorial health services in the remaining cases. Regarding the latter, 36% of patients received HA infusions at home (4% by private nursing services and 32% by public nursing services) and 8% at a territorial day-service.

The median weekly HA dose was 40 g, with 71% of patients receiving 40 g/week, 8% receiving a higher dose and 21% receiving a lower dose. Half of patients had a loading dose of HA: specifically, 30% received 40 g twice a week for 2 consecutive weeks according to the ANSWER study regimen, while the remaining 20% had a variable loading dose. The frequency of HA administration was once a week in most cases (59%), while it was more than once a week in about a third (32%) and less than once a week in only 9%.

Furthermore, the weekly dose and regimen changed in 36% of patients, two-thirds of whom had their dose gradually reduced due to clinical improvement, while the remaining one-third had their dose increased due to lack of response. Only a few patients (3%) reduced their weekly dose owing to logistical issues.

**Fig. 1:**
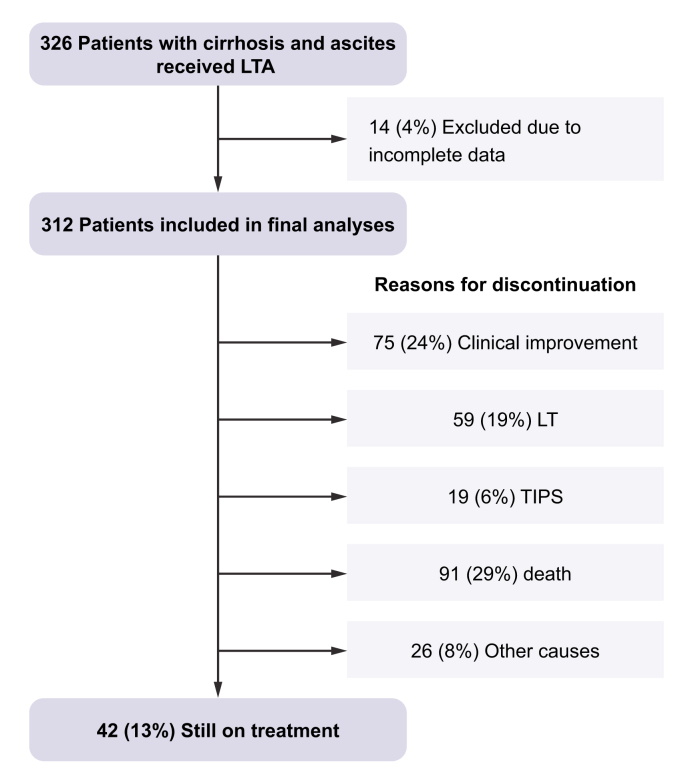
Patients included in the study and reasons for LTA treatment interruption. Median treatment duration was 9 (5-16) months in patients who stopped LTA due to clinical improvement, 10 (6-12) months in patients who received LT, 6 (3-13) months in patients who received TIPS, 6 (3-11) months in patients who stopped for other reasons and 11 (5-16) months in patients who died during LTA. LT, liver transplantation; LTA, long-term albumin; TIPS, transjugular intrahepatic portosystemic shunt.

Finally, the median duration of albumin treatment was 10 months (IQR 5–17). LTA was discontinued in 75 (24%) patients due to clinical improvement as judged by the physician, LT in 59 (19%) and TIPS in 19 (6%). Ninety-one (29%) patients died during LTA, while treatment was stopped in 23 (7%) due to the activation of palliative care. Only 3 (1%) patients discontinued LTA for logistical reasons. Finally, 42 (13%) patients were still on treatment at the end of the observational period (Fig. 1).

### Management of ascites and predictors of response

**Fig. 2:**
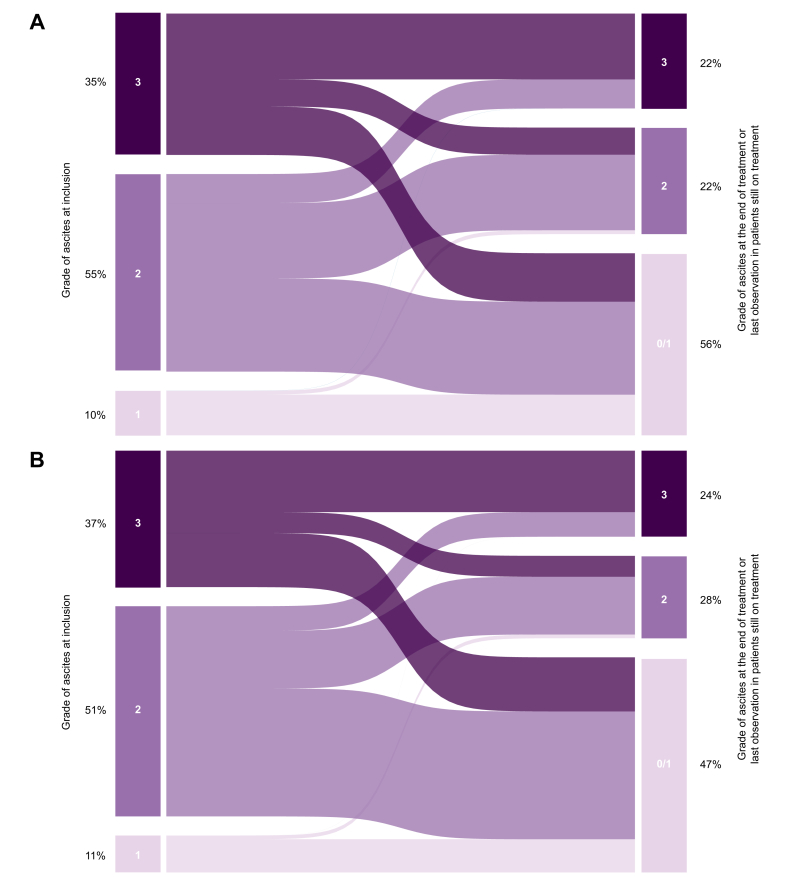
Sankey plots showing the patients with grade 1, 2 and 3 ascites at inclusion and those with grade 0-1, 2 and 3 ascites at the last observation before the end of treatment for any reason or at the last visit for those still on treatment on February 28^th^, 2023. (A) The whole study population (N = 312) and (B) the subgroup of patients who did not receive any aetiological treatment for cirrhosis during long-term albumin treatment or in the year prior to enrolment (n = 198).

At the time of the last observation before treatment interruption due to any cause or at the end of follow-up in patients still on treatment, the grade of ascites was significantly improved. Among patients with grade 3 ascites at inclusion, 44 (40%) improved to grade 0/1 ascites, while 17 (15%) improved to grade 2. Furthermore, 104 (61%) patients with grade 2 ascites at inclusion improved to grade 0/1 ascites. Finally, at the last assessment, 175 (56%) patients presented grade 0/1, 67 (22%) grade 2 and 70 (22%) grade 3 ascites (Fig. 2A). The median length of treatment for those who achieved grade 0/1 ascites was 11 (6-19) months.

**Fig. 3:**
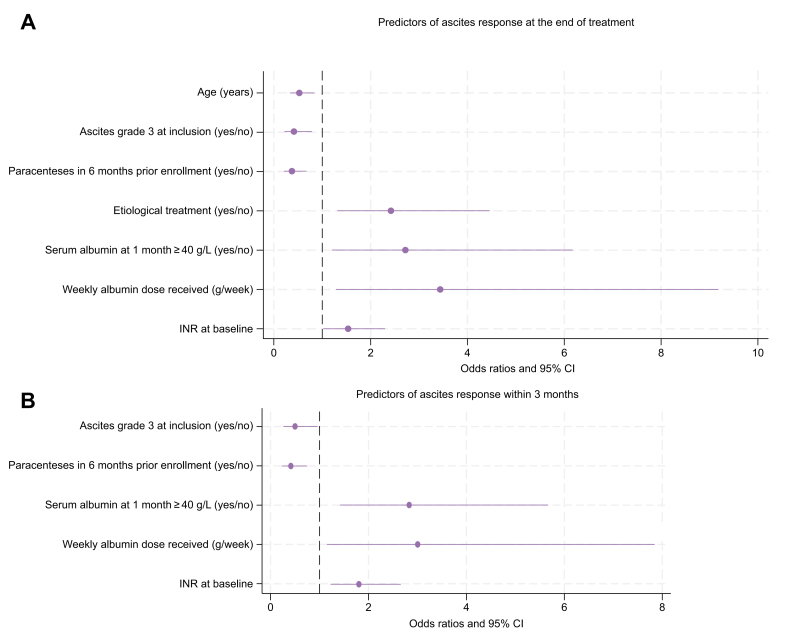
Independent predictors of ascites resolution. (A) Independent predictors of ascites resolution at the end of treatment or at last available visit in patients still on treatment at the end of follow-up. (B) Independent predictors of ascites resolution within 3 months from study inclusion. Odds ratio and 95% CI were estimated from multivariable logistic regression performed on 100 imputation datasets. Values from continuous variables were divided by the IQR except for albumin dose which was divided by the median.

We then searched for predictors of ascites resolution at the end of treatment or last observation in patients still on treatment at the end of follow-up. The comparison of clinical and treatment-related factors between patients achieving or not grade 0/1 at the univariate analysis is reported in Table S1. At the following multivariable logistic regression analysis, independent positive or negative predictors of response were age (OR 0.526; 95% CI 0.331–0.837; *p =* 0.007), grade 3 ascites (OR 0.414; 95% CI 0.219–0.786; *p =* 0.007), need for paracentesis in the 6 months prior to enrolment (OR 0.373; 95% CI 0.207–0.674; *p =* 0.001), aetiological treatment in the past 12 months or during LTA (OR 2.419; 95% CI 1.312–4.460; *p =* 0.005), weekly albumin dose (OR 3.436; 95% CI 1.286–9.182; *p =* 0.014), serum albumin concentration ≥40 g/L after 1 month of treatment (OR 2.717; 95% CI 1.196–6.173; *p =* 0.017) and international normalised ratio (INR) at baseline (OR 1.523; 95% CI 1.013–2.288; *p =* 0.043) (Fig. 3A, Table S2).

To further explore the role of aetiological treatment, we assessed the response of ascites to LTA in the 198 patients who did not receive any aetiological treatment for cirrhosis during LTA or in the year prior to enrolment. Results were similar to those found in the whole cohort: at baseline, 11% had grade 1 ascites, 51% grade 2 and 37% grade 3, whereas, at the end of treatment or at the last observation, 47% showed resolution of ascites (grade 0/1), 28% grade 2 and 24% grade 3 (Fig. 2B).

**Fig. 4:**
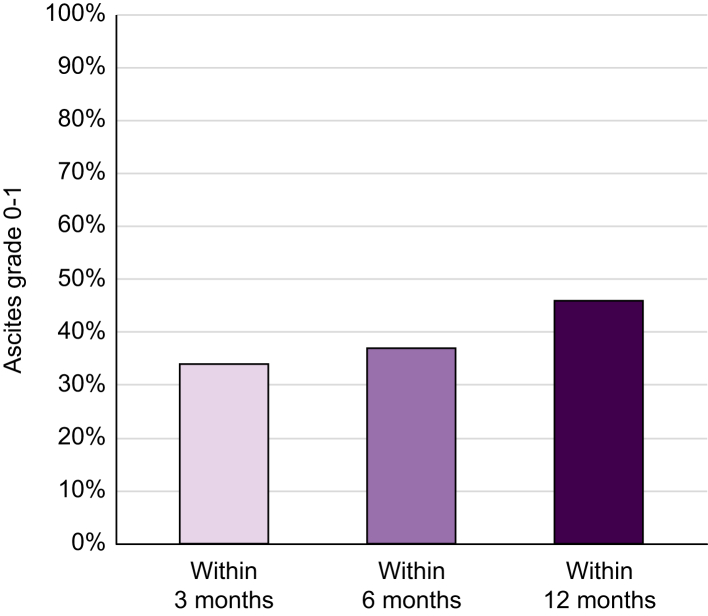
Percentage of patients with ascites grade 0/1 at 3 months, 6 months, and 12 months after initiation of long-term albumin treatment.

Among responders, ascites resolution occurred within a few months after the start of LTA in many cases. Indeed, ascites resolved in 34% of patients within the first 3 months of treatment, in 37% within 6 months and in 46% within 12 months (Fig. 4). It is worth noting that 92% of patients resolving ascites at 3 months maintained the response until the end of treatment.

We therefore investigated the factors predicting this early response to LTA. Table S3 reports the univariate analysis comparing clinical and treatment-related features of patients who resolved (n = 106) or not (n = 206) ascites within 3 months. At the multivariable analysis, independent positive or negative predictors of early response were grade 3 ascites at inclusion (OR 0.499; 95% CI 0.259–0.963; *p =* 0.038), need for paracentesis in the 6 months prior to inclusion (OR 0.414; 95% CI 0.229–0.749; *p =* 0.004), weekly dose of albumin administrated (OR 3.004; 95% CI 1.150–7.846; *p =* 0.025), serum albumin concentration ≥40 g/L after 1 month of treatment (OR 2.831; 95% CI 1.415–5.664; *p =* 0.003), and INR at baseline (OR 1.807; 95% CI 1.224–2.659; *p =* 0.003) (Fig. 3B, Table S4).

### Subgroup of patients with refractory ascites at inclusion

At the time of inclusion, 83 (27%) patients had refractory ascites according to ICA criteria. Of these, 27 had received less than 4 LVPs in the 6 months prior to starting LTA while 56 had received ≥4 LVPs.

At the end of treatment (or last available observation), 22 (26%) of the patients with refractory ascites at inclusion resolved ascites to grade 0/1. Of these, 13 patients discontinued LTA treatment due to clinical improvement, 6 were still on treatment, 2 died and 1 underwent transplantation after resolution of ascites. Moreover, 13 out of 22 patients (59%) who resolved ascites received an effective treatment for the aetiological cause of cirrhosis during LTA. Of the remaining 61 patients whose ascites did not resolve during LTA, 14 underwent TIPS placement, 26 died, 6 were transplanted, 9 were still on treatment, and 6 discontinued LTA for other reasons.

Overall, the median duration of treatment in patients with refractory ascites was 12 months (IQR 5-19). LTA was also associated with a reduction in the need for LVPs. As shown in Fig. S2, in the group of patients receiving less than 4 LVPs during the 6 months prior to inclusion in the study, 44% had no additional LVPs in the first 3 months of LTA and 59% between month 3 and 6 of LTA, while in those receiving at least 4 LVPs, 13% had no additional LVP in the first 3 months of LTA and 29% between month 3 and 6 of LTA.

### Subgroup of patients who discontinued treatment because of clinical improvement

**Fig. 5:**
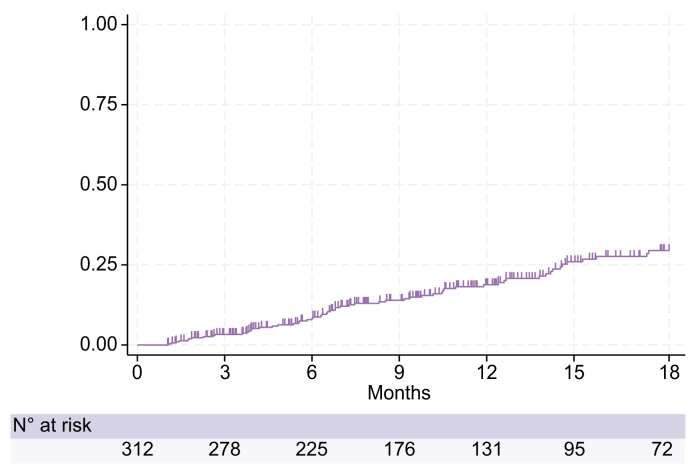
Cumulative incidence of treatment discontinuation due to clinical improvement up to 18 months. Censored patients are those who discontinued treatment for other reasons (death, LT, TIPS, other causes) or who interrupted follow-up before 18 months. The cumulative incidence was estimated according to the Kaplan-Meier method. LT, liver transplantation; TIPS, transjugular intrahepatic portosystemic shunt.

Seventy-five patients discontinued LTA treatment because of clinical improvement according to clinical judgement (Fig. 5). Severity of ascites, laboratory data and prognostic scores of these patients at baseline and at the end of treatment are reported in Table 2. Remarkably, 93% of patients had ascites grade 0/1 at discontinuation and a significant improvement of almost all laboratory data and prognostic scores was observed. Finally, only 9 (12%) patients resumed treatment after a median interruption of 15 (IQR 4-20) months due to grade 2-3 ascites recurrence.

**Table 2:** Characteristics of patients who discontinued long-term albumin treatment for clinical improvement (n = 75) at inclusion in the study and at the end of treatment.

	Inclusion	End of treatment	*p* value
**Ascites**			
Grade of ascites, n (%)			0.001
Ascites grade 0-1	5 (7)	70 (93)	
Ascites grade 2	45 (60)	5 (7)	
Ascites grade 3	25 (33)	0 (0)	
Refractory ascites	14 (19)	0 (0)	0.001

**Laboratory data**			
Hb (g/dl)	10.9 (9.8–12.8)	12.0 (10.7–13.1)	0.001
Sodium (mmol/L)	136 (133–139)	138 (136–140)	0.001
Bilirubin (mg/dl)	2.2 (1.0–4.0)	1.1 (0.8–1.9)	0.001
Creatinine (mg/dl)	0.9 (0.7–1.1)	1.0 (0.7–1.1)	0.777
Albumin (g/L)	31 (27–35)	39 (35–44)	0.001
INR	1.4 (1.2–1.6)	1.2 (1.2–1.3)	0.001

**Prognostic scores**			
Child-Pugh score	9 (7–10)	7 (6–8)	0.001
MELD score	14 (11–18)	10 (9–13)	0.001
MELD-Na score	18 (14–21)	13 (11–15)	0.001

Hb, haemoglobin; INR, international normalized ratio; MELD, model for end-stage liver disease; MELD-Na, model for end-stage liver disease incorporating serum sodium.

Data are reported by median and interquartile range or absolute frequency and percentage (%) as appropriate. Comparisons were performed by marginal homogeneity test and Wilcoxon signed-rank test as appropriate.

The baseline characteristics of patients stopping LTA due to clinical improvement compared with the other patients in the cohort are shown in Table S5. Specifically, these patients were younger, more frequently had an alcohol-related aetiology, and less severe portal hypertension (*i.e.*, milder degree of oesophageal varices, less frequent prior gastrointestinal bleeding and HE episodes, and higher platelet count). No statistical differences were observed regarding the prognostic scores, the grade of ascites and the prevalence of refractory ascites, although the latter was lower in patients who discontinued treatment because of clinical improvement.

The duration of albumin treatment was slightly but not significantly shorter in patients who discontinued LTA for clinical improvement (9 months [IQR 5-18] *vs.* 11 months [IQR 5-18], *p =* 0.35), while the median weekly albumin dose was 40 g in both groups (*p =* 0.09). Furthermore, aetiological treatment in the 12 months prior to enrolment or during LTA was more frequent in patients who discontinued treatment because of clinical improvement (59% *vs.* 30%, *p* <0.001). Finally, 9 patients (3% of the total cohort) fully met the Baveno VII "recompensation" criteria (in particular, they discontinued diuretic therapy).^8^

## Discussion

The present multicentre retrospective study describes for the first time the real-life experience on LTA administration in a large cohort of patients with cirrhosis and ascites. The main findings of this study can be summarised as follows: 1) LTA on top of diuretic therapy for the management of ascites is feasible and safe, with very high patient compliance; 2) ascites resolution occurred in about half of patients, including some with a previous diagnosis of refractory ascites, 3) several clinical- and treatment-related factors can predict the response of ascites to LTA, providing useful information for personalizing treatment; 4) the duration of LTA was less than 1 year in the majority of cases, with less than 15% still on treatment after 2 years; and 5) almost a quarter of patients were able to discontinue treatment due to clinical improvement.

The first main result of the present study assessing LTA in daily clinical practice is that the adherence to treatment is extremely high, as only 1% of patients discontinued treatment due to logistic issues and only 5% were lost to follow-up during treatment, mostly during the initial waves of the COVID pandemic, supporting the assumption that LTA is perceived by patients as beneficial for their health. Several modalities of LTA treatment used in the ANSWER trial have been translated into daily clinical practice, such as the median weekly dose of albumin, which was indeed 40 g/L, and the setting of albumin infusion, which was performed in territorial services or even at home in almost 50% of patients. However, it appears clear that physicians tried to tailor treatment at the individual level in many cases by modifying the loading dose at the beginning of LTA and changing the weekly dose based on the patient’s clinical needs. Finally, although retrospective studies are not the ideal approach to assessing safety, no serious adverse events requiring discontinuation of albumin, such as allergic reactions or fluid overload, have been reported. Of note, about half of the study population had comorbidities and 17% had chronic heart disease.

A second major finding of the present study is the efficacy of LTA for managing ascites in real-world clinical practice, supporting the findings of the ANSWER trial.^1^ However, this real-life study adds some important novel information, providing more details on the target population, the modalities of treatment and the possibility of stopping treatment.

The optimal candidates for treatment, with the highest probability of ascites resolution, appear to be those with clinically evident ascites despite a moderate diuretic dose and without the need for paracentesis in the previous 6 months. Nonetheless, in clinical practice, LTA is also prescribed in patients with more severe ascites, including those with an established diagnosis of refractory ascites, who were excluded from the ANSWER trial.^1^ Interestingly, in about 25% of patients with a diagnosis at inclusion of refractory ascites according to the ICA criteria, ascites resolved to grade 0-1 and the need for LVPs was markedly reduced, in line with the results reported by a monocentric prospective, non-randomised trial from the Padua group^9^ and by a recent small retrospective cohort from Australia.^10^

Another important finding is that approximately one-third of patients achieved grade 0/1 ascites within 3 months, although a consistent number of additional patients may become responders later. Interestingly, more than 90% of these “early” responders maintained the response until end of treatment. Analysis of the factors predicting response provides useful insights on how to translate this data into the decision-making process.

Besides the severity of ascites, two treatment-related factors were independently associated with its resolution: the dose of the albumin infused, and the serum albumin concentration achieved at 1 month of treatment. This result confirms the role of the on-treatment serum albumin concentration as a useful tool for tailoring treatment at the individual level, as already demonstrated by a *post hoc* analysis of the ANSWER trial^11^ and supported by other indirect evidence.^12^ Thus, increasing the dose of albumin, at least transiently, to achieve a target serum albumin concentration close to 40 g/L may represent a possibility for patients with an insufficient response after the first weeks of therapy. Interestingly, in this study, 44% of patients who had their HA increased due to unsatisfactory response achieved grade 0/1 ascites at the end of LTA treatment (data not shown). Finally, a longer baseline INR was also identified as a predictor of ascites resolution. Although statistically significant, this unexpected result is unlikely to be clinically relevant, as the difference between the two groups is minimal (1.3 *vs.* 1.4, Table S1).

Another factor to take into account for predicting response is the possibility of removing the underlying cause of cirrhosis (*i.e*., achieving alcohol abstinence or suppressing/eradicating hepatic viruses), which is undoubtedly the most effective disease-modifying treatment in patients with decompensated cirrhosis, although the time required to unveil the benefits can be quite long.[13], [14], [15], [16] In the present study, a recent or concomitant effective aetiological treatment independently predicted ascites resolution, but not within 3 months, and was associated with LTA discontinuation due to clinical improvement. Besides emphasizing once again the importance of eliminating the underlying cause of cirrhosis,^13^ this result might suggest that LTA may be prolonged in these patients, even in the presence of an unsatisfactory response of ascites within 3 months, with the purpose of contributing to clinical stabilization until the benefits of aetiological treatment become manifest.

The present investigation also provides important information to address a major open issue about LTA, that is its duration and the possibility of stopping treatment.

First, the median duration of LTA was 10 months, while less than 15% of patients received albumin for more than 2 years, and less than 10% were still on treatment at the end of follow-up. Thus, physicians and healthcare policymakers should expect that, once started, LTA will last no longer than 12 months in more than half of patients and no longer than 24 months in most cases.

Second, even more importantly, almost one out of four patients can interrupt LTA due to clinical improvement, with a very low probability (about 10%) of resuming treatment because of ascites recurrence. As expected, these patients are younger, have more alcohol-related cirrhosis, and more frequently receive an effective aetiological treatment. Interestingly, however, they also present clinical evidence of milder portal hypertension, but a similar severity of prognostic scores and degree of ascites. Potential clinical factors which should prompt physicians to stop LTA are represented by the persistent resolution of ascites to grade 0/1 and the significant reduction of MELD and MELD-Na scores. Interestingly, in this real-world study, a reduction in the dose and/or frequency of albumin administration was performed in many patients before stopping LTA because of clinical improvement.

The present study has several limitations. First, this is a retrospective study with the inherent limitations of this type of clinical investigation. With this in mind, we have decided to limit our analysis to a few solid data, avoiding the collection of more granular data, such as the changes during follow-up of concomitant therapies, including the diuretic doses. In this regard, however, patients were managed by experienced hepatologists who likely prescribed diuretic therapy at the optimal dose allowed by the patient's renal function and hemodynamic status. Furthermore, all the centres involved participated in the ANSWER trial and have great experience in clinical research. Second, detailed alcohol follow-up (including biomarkers of alcohol consumption) was not available, so precise changes in consumption patterns were not available. Only patients who have achieved prolonged alcohol abstinence with certainty by the referring physician were included among those effectively treated. However, it should be recognised that a reduction in alcohol consumption (even without prolonged abstinence) could be beneficial in the control of ascites. Third, except for ascites, the efficacy of LTA on other clinical outcomes was not assessed. However, this was not the major objective of our study, which instead aimed to analyse how the results of the ANSWER trial have been translated into clinical practice and to provide practical information that are not usually addressed by RCTs. Moreover, the analysis of the efficacy on many clinical outcomes implies the comparison with a control group. As LTA has become standard of care in Italian hepatology centres, it would have been very difficult to find enough patients receiving HA only for the evidence-based indications to build up a comparable control group. In addition, the results of a large multicentre, open-label RCT investigating the “effects of long-term administration of human albumin on subjects with decompensated cirrhosis and ascites” (PRECIOSA study; NCT03451292), which enrolled more than 400 patients in Europe and the US with 1-year transplant-free survival as the primary endpoint, are expected at the end of 2024, and will provide a more complete assessment of efficacy than any retrospective study. Fourth, LTA is scarcely used outside Italy due to scientific scepticism and logistical or economic issues. Thus, one can argue that these data cannot be extended to other countries. However, while waiting for the results of the PRECIOSA trial, we believe that this study represents a unique opportunity to share the real-life experience of hepatological centres where LTA is a frequently used option for the medical management of ascites, thus providing novel information to address the aforementioned concerns. Lastly, we were not able to collect the exact number of patients who were not offered treatment due to expected poor adherence or who refused treatment. However, all centres estimated that these cases accounted for less than 10% of the entire cohort. Of note, 38 (12%) patients included in the analysis presented with social problems.

In conclusion, the Real-ANSWER study indicates that the best candidates for LTA are patients with cirrhosis and persistent clinically manifest (grade 2/3) ascites despite a moderate dosage of diuretics. However, patients with refractory ascites according to the ICA criteria cannot be excluded *a priori*, particularly if the refractoriness has been diagnosed recently or elsewhere and/or patients are excluded from TIPS or amenable to an effective aetiological treatment. The application of LTA in daily clinical practice is feasible, without safety concerns related to the risk of pulmonary oedema or severe allergic reactions, and represents a valid treatment option in patients with decompensated cirrhosis by facilitating the management of ascites when given on top of diuretics. Rather than an alternative therapy, LTA should be integrated with the other treatments available for patients with difficult-to-treat ascites. The factors predicting response to LTA identified in the present study can help physicians to tailor LTA at the individual level and to optimise the decision-making process.

## Abbreviations

HA, human albumin; HCC, hepatocellular carcinoma; HE, hepatic encephalopathy; HRS, hepatorenal syndrome; ICA, International Club of Ascites; INR, international normalised ratio; LT, liver transplantation; LTA, long-term albumin; LVP, large volume paracentesis; MAP, mean arterial pressure; MELD, model for end-stage liver disease; MELD-Na, model for end-stage liver disease incorporating serum sodium; MICE, multiple imputation chained equations; RCT, randomised-controlled trial; SBP, spontaneous bacterial peritonitis; TIPS, transjugular intrahepatic portosystemic shunt.

## Financial support

The present study was supported by an unrestricted grant “Investigator Sponsored Research” by Grifols SA. The Funder had no role in the design and conduct of the study and in the collection, analysis, and interpretation of the data, decision to publish or in the preparation of the manuscript.

## Authors’ contributions

EP, GZ, GI, MB, PC: study concept and design, interpretation of data, drafting of the manuscript; EP, GI, CDV, GT, MT, RG, DB, AL, SG: collection of data; EP, MB, GB: analysis of data; MB, GZ, SP, PA, PT, VC, MD, VDM, SN, PC: critical revision for important intellectual content.

## Data availability statement

The data supporting the results of this study are available from the corresponding author upon reasonable request. The data are not publicly available due to privacy/ethical restrictions.

## Ethics approval statement

The study protocol was approved by the local institutional review boards at each participating centre.

## Conflict of interest

The following authors disclose conflicts of interests: GZ: Grifols SA (speaking bureau). SP: Plasma Protein Therapeutics Association, Boehringer Ingelheim, Resolution therapeutics (Consultant); Grifols SA and MEDSCAPE (Sponsored lectures). MT: Gilead and Grifols (travel support). PA: Biovie (advisory board and patent), CSL Behring (speaker invitation and travel grant), Grifols (speaker invitation), Kedrion (speaker invitation), Biomarin (advisory board), GenFit SA (advisory board). PC: Grifols SA (speaking bureau and research grant), Octapharma SA (speaking bureau and research grant), CSL Behring (speaking bureau and advisory boards), Gilead (speaking bureau). All the other authors had no conflicts of interest.

Please refer to the accompanying ICMJE disclosure forms for further details.
