# Supplementary material for: Real-world experience with long-term albumin in patients with cirrhosis and ascites
Source: JHEP Rep. 2024 Sep 17;6(12):101221. doi: 10.1016/j.jhepr.2024.101221 (PMC11617394; doi:10.1016/j.jhepr.2024.101221)
Supplement: Multimedia component 1 [file mmc1.pdf]

Real-world experience with long-term albumin in patients with cirrhosis and ascites

Enrico Pompili, Giacomo Zaccherini, Salvatore Piano, Pierluigi Toniutto, Antonio Lombardo, Stefania Gioia, Giulia Iannone, Clara De Venuto, Marta Tonon, Roberta Gagliardi, Maurizio Baldassarre, Greta Tedesco, Giorgio Bedogni, Marco Domenicali, Vito Di Marco, Silvia Nardelli, Vincenza Calvaruso, Davide Bitetto, Paolo Angeli, Paolo Caraceni

Table of contents

Multiple imputation by chained equations (MICE)....................2

Fig. S1 3

Fig. S2 4

Table S1. 5

Table S2 8

Table S3. 9

Table S4 12

Table S5. 13

Multiple imputation by chained equations (MICE)

We used MICE to create 100 complete versions of data by replacing missing values with plausible data values (1, 2).

The imputed continuous variables were albumin, creatinine, INR and haemoglobin at baseline and at 30 days from inclusion. The complete variables in the regression model were center (discrete), sex (discrete), age (continuous), etiology of cirrhosis (discrete), history of ascites (discrete), history of HCC (discrete), history of type 2 diabetes mellitus (discrete), inclusion in liver transplantation waitlist (discrete), previous hepatic encephalopathy (discrete), previous spontaneous bacterial peritonitis (discrete), previous gastrointestinal bleeding (discrete), etiological treatment performed in the 12 months prior enrolment or during the follow-up (discrete).

Imputation was performed using linear regression on 100 MI datasets. Trace plots of imputed values against iteration numbers were used to assess the stationarity of the chains. The imputer and the analyst were the same person, and the scope of the MI model was narrow, i.e., it was devised for testing only the present study hypothesis (3).

The linearity of association of the outcomes with age was tested using multivariable fractional polynomials for multiple imputation (4). The association was always linear. Logistic regression of MI datasets was used to determine the multivariable associations between:

i) Early ascites resolution and: age, etiology of cirrhosis, grade of ascites, history of paracentesis in the 6 months prior enrolment, previous etiological treatment, baseline albumin, creatinine, INR, bilirubin, haemoglobin, 1-month albumin ≥ 40g/L, presence of medium-high risk oesophageal varices, albumin dose.

ii) Resolution of ascites at the end of treatment or last observation in patients still on treatment at the end of follow-up with: age, etiology of cirrhosis, grade of ascites, history of paracentesis in the 6 months prior enrolment, previous or concomitant etiological treatment, baseline albumin, creatinine, INR, bilirubin, haemoglobin, 1-month albumin ≥ 40g/L, presence of medium-high risk oesophageal varices, albumin dose.

References

1. van Buuren S. Flexible Imputation of Missing Data. Chapman & Hall/CRC; 2018:416.
2. White IR, Royston P, Wood AM. Multiple imputation using chained equations: Issues and guidance for practice. Stat Med. 2011;30:377-399. 10.1002/sim.4067
3. Sterne JA, White IR, Carlin JB, Spratt M, Royston P, Kenward MG, Wood AM, Carpenter JR. Multiple imputation for missing data in epidemiological and clinical research: potential and pitfalls. BMJ. 2009;338:b2393. 10.1136/bmj.b2393
4. Morris TP, White IR, Carpenter JR, Stanworth SJ, Royston P. Combining fractional polynomial model building with multiple imputation. Stat Med. 2015;34:3298-3317. 10.1002/sim.6553

Fig. S1

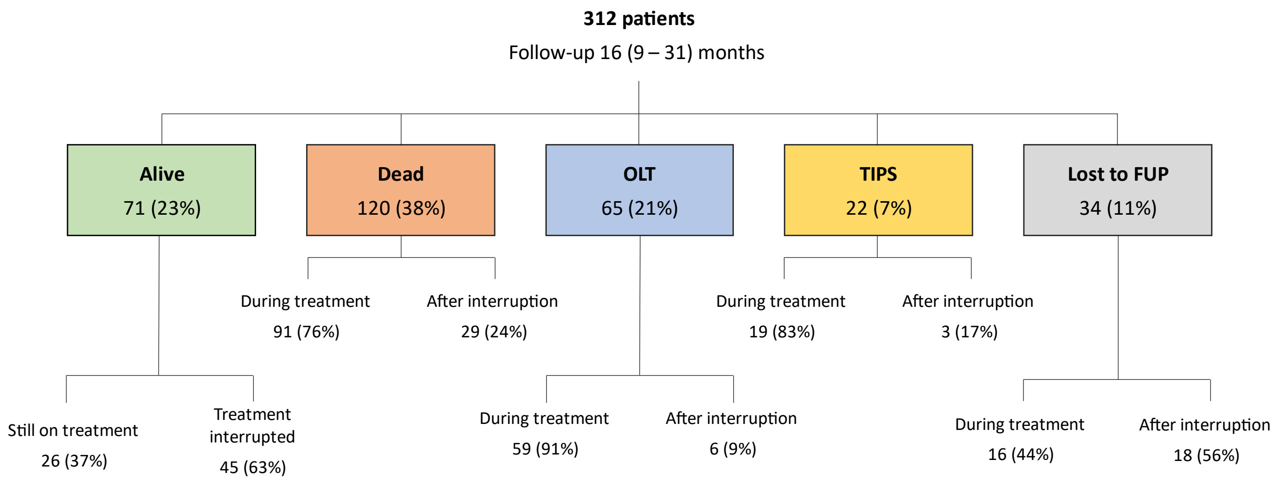

Patients' outcome at the end of follow up. During follow-up until February 28th 2023 (median length was: 16 months [IQI 9 – 31]), 120 (38%) died. Of these patients, 91 subjects died while long-term treatment was ongoing (or had recently stopped treatment to receive palliative care), while 29 died several months after treatment had already been stopped. Of the remaining 192 patients, 23% was alive with 8% still on HA treatment, 21% had a liver transplant, 7% received a TIPS and 11% were lost to follow-up.

Fig. S2

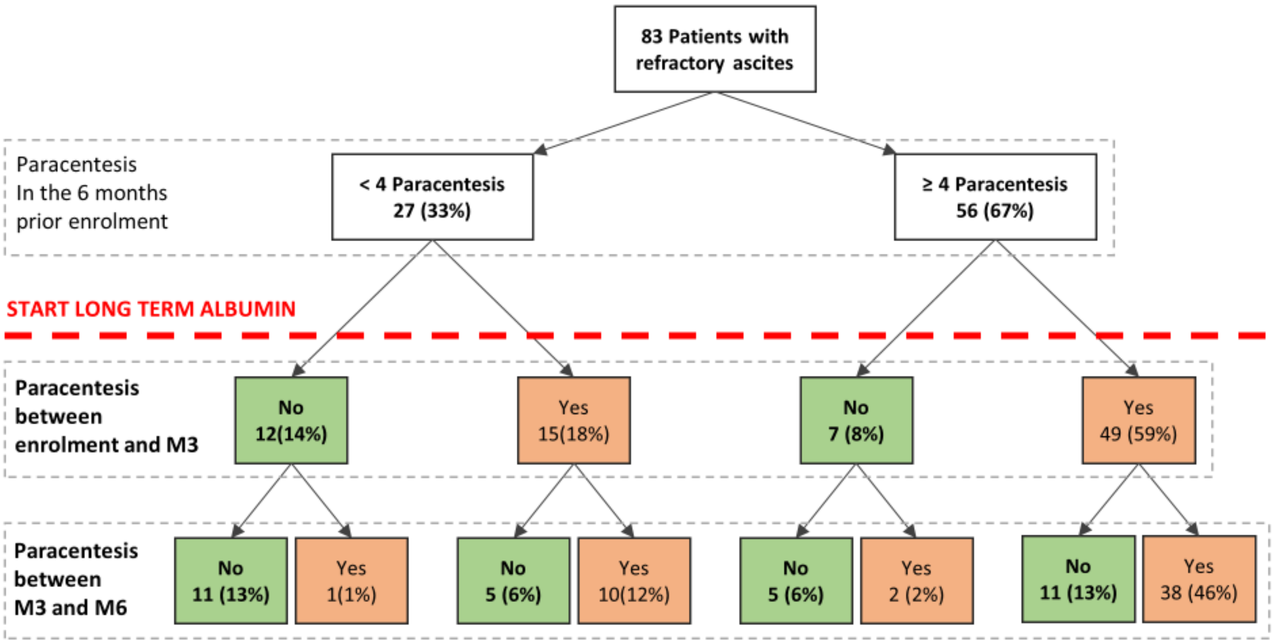

Paracentesis in the subgroup of patients with diagnosis of refractory ascites at inclusion. Analyses of the subgroup of patients with refractory ascites according to the *International Club of Ascites* criteria ad enrolment (n=83): patients who underwent large-volume paracentesis (LVP) in the 6 months prior to starting long-term albumin treatment and did or did not require further LVP in the first 3 months of LTA and between the 3rd and 6th month of LTA.

Reference:

Kevin P. Moore, Fl. Wong, P. Gines, M. Bernardi, A. Ochs, F. Salerno, et al., The management of ascites in cirrhosis: Report on the consensus conference of the International Ascites Club, Hepatology, 2003;38:258-266. doi.org/10.1053/jhep.2003.50315

Table S1. Comparison between patients who achieved ascites resolution (grade 0-1) at the end of treatment or at last observation in patients still on treatment at the end of follow-up.

	**No resolution** N = 137	**Resolution** N = 175	**p**
**Demographic data**			
Age (years)	65 (59 – 73)	60 (53 – 68)	0.001
Male sex (n, %)	104 (76)	115 (66)	0.051
**Etiology of cirrhosis**			0.013
Alcohol (n, %)	42 (31)	74 (42)	
MASLD (n, %)	30 (22)	18 (10)	
Viral (n, %)	22 (16)	23 (13)	
Alcohol+Viral (n, %)	9 (7)	23 (13)	
Alcohol+MASLD (n, %)	21 (15)	19 (11)	
Other (n, %)	13 (10)	18 (10)	
**Ascites**			
Grade of ascites			0.001
- Ascites grade 0–1 (n, %)	3 (2)	27 (15)	
- Ascites grade 2 (n, %)	67 (49%)	104 (59)	
- Ascites grade 3 (n, %)	67 (49%)	44 (25)	
Refractory ascites (n, %)	61 (45)	22 (13)	0.001
Previous paracentesis within 6 months prior to enrollment (n of patients, %)			0.001
- 1–3 paracentesis in last 6 months (n of patients, %)	38 (28)	45 (26)	
- ≥ 4 paracenteses in last 6 months (n of patients, %)	50 (37)	14 (8)	
**Medical History**			
Presence of esophageal varices			0.001
- Low risk of bleeding	58 (43)	110 (63)	
- Moderate/High risk of bleeding	40 (29)	26 (15)	
Previous overt HE (n, %)	42 (31)	51 (29)	0.772

Previous gastrointestinal bleeding (n, %)	31 (23)	29 (17)	0.178
Previous spontaneous bacterial peritonitis (n, %)	16 (12)	14 (8)	0.274
Previous hepato–renal syndrome (n, %)	8 (6)	14 (8)	0.443
HCC within Milan criteria (n, %)	16 (12)	16 (9)	0.464
Active awaiting list for LT (n, %)	16 (12)	25 (14)	0.499
Active alcohol consumption (n, %)	13 (18)	34 (29)	0.083
Etiological treatment (<12 month or during the treatment) (n, %)	33 (24)	81 (46)	0.001
**Concomitant medications**			
Antialdosteronic – daily dosage (mg)	200 (100–200)	200 (100–300)	0.238
Furosemide – daily dosage (mg)	50 (25–75)	50 (25–75)	0.519
Non–Selective beta–blockers (n, %)	73 (53)	87 (50)	0.531
Rifaximin (n, %)	49 (36)	73 (42)	0.285
Fluoroquinolones for SBP prophylaxis (n, %)	4 (3)	7 (4)	0.608
**Comorbidities**			
Chronic Heart Disease (n, %)	27 (20)	27 (15)	0.321
Chronic kidney disease (n, %)	27 (20)	18 (10)	0.020
Chronic lung disease (n, %)	16 (12)	14 (8)	0.274
Neurologic diseases (n, %)	4 (3)	8 (5)	0.452
Diabetes (n, %)	53 (39)	57 (33)	0.262
**Laboratory and hemodynamic data at inclusion**			
Hb (g/dL)	10.2 (9.2–12.0)	10.9 (9.7–12.5)	0.059
WBC (10^9/L)	5.3 (4.0–7.0)	5.3 (3.8–7.3)	0.849
Platelets (10^9/L)	102 (72–144)	103 (62–143)	0.575
Sodium (mmol/L)	136 (133–139)	136 (133–139)	0.247
Bilirubin (mg/dL)	1.6 (1.1–3.3)	2.5 (1.4–4.2)	0.005
Creatinine (mg/dL)	1.1 (0.8–1.4)	0.9 (0.7–1.1)	0.001
Albumin (g/L)	31 (28–35)	31 (27–35)	0.788
INR	1.3 (1.2–1.5)	1.4 (1.3–1.6)	0.005
MAP (mmHg)	83 (80–90)	87 (77–93)	0.438

HR (bpm)	72 (68–80)	75 (68–83)	0.111
**Prognostic scores**			
Child–Pugh score	8 (7–10)	9 (7–10)	0.447
MELD score	14 (11–18)	15 (12–18)	0.161
MELD–Na score	18 (14–21)	18 (15–21)	0.334

Data is reported by median and interquartile range or absolute frequency and percentage (%) as appropriate. Comparisons between groups were performed by means of Student's t test, Mann–Whitney U test or the chi2 test when appropriate. Abbreviations: MASLD: metabolic dysfunction-associated steatotic liver disease; HE: hepatic encephalopathy; HCC: hepatocellular carcinoma; LT: liver transplantation; NSBB: nonselective beta blockers; SBP: spontaneous bacterial peritonitis; Hb: haemoglobin; WBC: white blood cells; INR: International Normalized Ratio; MAP: mean arterial pressure; HR: heart rate; MELD: Model for End-stage Liver disease; MELD-Na: Model for End-stage Liver disease incorporating serum sodium.

Table S2. Multivariable logistic regression analysis of predictors of ascites resolution (grade 0-1) at the end of treatment or at last observation in patients still on treatment at the end of follow-up.

	OR	95% CI		p
Age (Years)	0.526	0.331	0.837	0.007
Grade 3 ascites at baseline (yes/no)	0.414	0.219	0.786	0.007
Paracentesis in the 6 months prior enrollment (yes/no)	0.373	0.207	0.674	0.001
Etiological treatment in the past 12 months or during LTA (Yes/no)	2.419	1.312	4.460	0.005
Serum albumin at baseline (g/L)	1.101	0.711	1.705	0.665
Serum albumin ≥ 40g/L at 1 month (g/L)	2.717	1.196	6.173	0.017
Creatinine at baseline (mg/dL)	0.849	0.551	1.308	0.459
INR at baseline	1.523	1.013	2.288	0.043
Bilirubin at baseline (mg/dL)	0.938	0.743	1.185	0.596
Haemoglobin at baseline (g/dL)	1.392	0.918	2.111	0.119
Medium/high risk oesophageal varices (yes/no)	0.577	0.284	1.171	0.128
Average weekly albumin dose received (gr/week)	3.436	1.286	9.182	0.014
Etiology of cirrhosis: alcohol	1.540	0.650	3.652	0.326
Etiology of cirrhosis: MASLD	0.702	0.251	1.963	0.501
Etiology of cirrhosis: other	1.287	0.421	3.935	0.657
Etiology of cirrhosis: alcohol+viral	1.359	0.421	4.381	0.607
Etiology of cirrhosis: alcohol+MASLD	0.515	0.174	1.525	0.231

Odds ratios (OR) and 95%CI from logistic regression performed on 100 imputation datasets. Missing values were 4 (1%) for serum albumin at baseline, 76 (25%) for serum albumin at 1 month, 2 (0.5%) for serum creatinine at baseline, 4 (1%) for INR at baseline, 3 (1%) for bilirubin at baseline and 1 (0.3%) for haemoglobin at baseline. The reference category for etiology of cirrhosis is the viral etiology. Severe ascites was compared with mild/moderate ascites. Values for continuous variables are divided by the IQI, except for albumin dose, which is divided by the median.

Table S3. Comparison between patients who achieved ascites resolution (grade 0-1) within three months from study inclusion.

	No resolution<br>N = 206	Resolution<br>N=106	p
**Demographic data**			
Age (years)	63 (56 - 71)	60 (53 - 68)	0.035
Male sex (n, %)	149 (72%)	70 (66%)	0.250
**Etiology of cirrhosis**			0.531
Alcohol (n, %)	71 (34)	45 (42)	
MASLD (n, %)	33 (16)	15 (14)	
Viral (n, %)	29 (14)	16 (15)	
Alcohol+Viral (n, %)	20 (10)	12 (11)	
Alcohol+MASLD (n, %)	29 (14)	11 (10)	
Other (n, %)	24 (12)	7 (7)	
**Ascites**			
Grade of ascites			0.001
- Ascites grade 1 (n, %)	24 (11%)	6 (6%)	
- Ascites grade 2 (n, %)	95 (46%)	76 (71%)	
- Ascites grade 3 (n, %)	87 (42%)	24 (23%)	
Refractory ascites (n, %)	73 (35%)	10 (9%)	0.001
Previous paracentesis within 6 months prior to enrollment (n of patients, %)	116 (56%)	31 (29%)	0.001
- 1-3 paracentesis in last 6 months (n of patients, %)	56 (27%)	27 (25%)	
- ≥ 4 paracenteses in last 6 months (n of patients, %)	60 (29%)	4 (4%)	
**Medical History**			
Presence of esophageal varices			0.061
- Low risk of bleeding	101 (49%)	67 (63%)	
- Moderate/High risk of bleeding	49 (24%)	17 (16%)	
Previous overt HE (n, %)	61 (30%)	32 (30%)	0.916

Previous gastrointestinal bleeding (n, %)	42 (20%)	18 (17%)	0.470
Previous spontaneous bacterial peritonitis (n, %)	18 (9%)	12 (11%)	0.464
Previous hepato-renal syndrome (n, %)	12 (6%)	10 (9%)	0.248
HCC within Milan criteria (n, %)	21 (10%)	11 (10%)	0.960
Active awaiting list for LT (n, %)	24 (12%)	17 (16%)	0.277
Active alcohol consumption (n, %)	27 (13)	20 (19)	0.185
Etiological treatment (<12 month before inclusion) (n, %)	44 (21)	33 (31)	0.058
**Medications at inclusion**			
Antialdosteronic – daily dosage (mg)	200 (100 - 200)	200 (100 - 300)	0.502
Furosemide – daily dosage (mg)	50 (25 – 75)	50 (25 – 75)	0.123
Non-Selective beta-blockers (n, %)	96 (47%)	56 (53%)	0.297
Rifaximin (n, %)	77 (37)	45 (43)	0.384
Fluoroquinolones for SBP prophylaxis (n, %)	8 (4)	3 (3)	0.633
**Comorbidities**			
Chronic Heart Disease (n, %)	37 (18%)	17 (16)	0.671
Chronic kidney disease (n, %)	33 (16)	12 (11)	0.256
Chronic lung disease (n, %)	20 (10)	10 (9)	0.938
Neurologic diseases (n, %)	7 (3)	5 (5)	0.566
Diabetes (n, %)	74 (36)	36 (34)	0.731
**Previous decompensation**			
Previous hepato-renal syndrome (n, %)	12 (6%)	10 (9%)	0.248
Previous gastrointestinal bleeding (n, %)	42 (20%)	18 (17%)	0.470
Previous spontaneous bacterial peritonitis (n, %)	18 (9%)	12 (11%)	0.464
Previous HE (n, %)	61 (30%)	32 (30%)	0.916
**Laboratory and hemodynamic data at inclusion**			
Hb (g/dL)	10.7 (9.6 - 12.3)	10.7 (9.2 - 12.2)	0.544
WBC (10^9/L)	5.4 (4.0 - 7.1)	4.9 (3.5 - 7.2)	0.307
Platelets (10^9/L)	103 (72 - 151)	98 (62 - 134)	0.103

Sodium (mmol/L)	136 (133 - 139)	137 (134 – 139)	0.340
Bilirubin (mg/dL)	1.9 (1.2-3.8)	2.5 (1.5-4.3)	0.097
Creatinine (mg/dL)	1.00 (0.80-1.28)	0.88 (0.71-1.10)	0.005
Albumin (g/L)	31 (27-35)	31 (27-35)	0.221
INR	1.35 (1.21-1.55)	1.50 (1.31-1.67)	0.001
MAP (mmHg)	85 (77 - 92)	87 (78 – 90)	0.415
HR (bpm)	75 (68 - 83)	72 (68 – 80)	0.475
**Prognostic scores**			
Child-Pugh score	8 (7 - 10)	8 (8 - 10)	0.436
MELD score	14 (11 - 17)	15 (12 - 18)	0.059
MELD-Na score	18 (14 - 21)	18 (15 - 21)	0.345

Data is reported by median and interquartile range or absolute frequency and percentage (%) as appropriate. Comparisons between groups were performed by means of Student's t test, Mann–Whitney U test or the chi2 test when appropriate. Abbreviations: MASLD: metabolic dysfunction-associated steatotic liver disease; HE: hepatic encephalopathy; HCC: hepatocellular carcinoma; LT: liver transplantation; NSBB: nonselective beta blockers; SBP: spontaneous bacterial peritonitis; Hb: haemoglobin; WBC: white blood cells; INR: International Normalized Ratio; MAP: mean arterial pressure; HR: heart rate; MELD: Model for End-stage Liver disease; MELD-Na: Model for End-stage Liver disease incorporating serum sodium.

Table S4. Multivariable logistic regression analysis of predictors of ascites resolution (grade 0-1) within three months from study inclusion.

	OR	95% CI		p
Age (years)	0.934	0.602	1.447	0.761
Grade 3 ascites at baseline (yes/no)	0.499	0.259	0.963	0.038
Paracentesis in the 6 months prior enrollment (yes/no)	0.414	0.229	0.749	0.004
Etiological treatment in the past 12 months (yes/no)	1.668	0.883	3.151	0.114
Serum albumin at baseline (g/L)	1.022	0.685	1.524	0.914
Serum albumin ≥ 40g/L at 1 month (g/L)	2.831	1.415	5.664	0.003
Creatinine at baseline (mg/dL)	0.867	0.554	1.358	0.535
INR at baseline	1.804	1.224	2.659	0.003
Bilirubin at baseline (mg/dL)	0.971	0.785	1.201	0.787
Haemoglobin at baseline (g/dL)	0.923	0.600	1.421	0.719
Medium/high risk oesophageal varices (yes/no)	0.854	0.413	1.767	0.671
Weekly albumin dose received (gr/week)	3.004	1.150	7.846	0.025
Etiology of cirrhosis: alcohol	0.982	0.424	2.271	0.967
Etiology of cirrhosis: NASH	0.755	0.279	2.040	0.581
Etiology of cirrhosis: other	0.375	0.115	1.227	0.105
Etiology of cirrhosis: alcohol+viral	0.610	0.199	1.863	0.386
Etiology of cirrhosis: alcohol+MASLD	0.393	0.131	1.172	0.094

Odds ratios (OR) and 95%CI from logistic regression performed on 100 imputation datasets. Missing values were 4 (1%) for serum albumin at baseline, 76 (25%) for serum albumin at 1 month, 2 (0.5%) for serum creatinine at baseline, 4 (1%) for INR at baseline, 3 (1%) for bilirubin at baseline and 1 (0.3%) for hemoglobin at baseline. The reference category for etiology of cirrhosis is the viral etiology. Severe ascites was compared with mild/moderate ascites. Values for continuous variables are divided by the IQI, except for albumin dose, which is divided by the median.

Table S5. Comparison of baseline characteristics of patients who interrupted long-term albumin treatment due to clinical improvement and patients who interrupted treatment for other reason or still on treatment at the end of follow up.

	**Other patients**	**Interruption for clinical improvement**	**p**
	N = 237	N= 75	
**Demographic data**			
Age (years)	64 (57-70)	60 (50-66)	0.001
Male sex (n, %)	169 (71)	50 (66)	0.444
**Etiology of cirrhosis**			0.001
Alcohol (n, %)	77 (33)	39 (52)	
MASLD (n, %)	46 (19)	2 (3)	
Viral (n, %)	34 (14)	11 (15)	
Alcohol+Viral (n, %)	22 (9)	10 (13)	
Alcohol+MASLD (n, %)	30 (13)	10 (13)	
Other (n, %)	28 (12)	3 (4)	
**Ascites**			
Grade of ascites			0.468
- Ascites grade 0-1 (n, %)	25 (11)	5 (7)	
- Ascites grade 2 (n, %)	126 (53)	45 (60)	
- Ascites grade 3 (n, %)	86 (36)	25 (33)	
Refractory ascites (n, %)	69 (29)	14 (19)	0.074
Previous paracentesis within 6 months prior to enrollment (n of patients, %)			0.236
- 1-3 paracentesis in last 6 months (n of patients, %)	66 (28%)	17 (23)	
- ≥ 4 paracenteses in last 6 months (n of patients, %)	52 (22)	12 (16)	
**Medical History**			
Presence of esophageal varices			0.014
- Low risk of bleeding	120 (51)	48 (64)	

- Moderate/High risk of bleeding	59 (25)	7 (9)	
Previous overt HE (n, %)	80 (34)	13 (17)	0.007
Previous gastrointestinal bleeding (n, %)	53 (22)	7 (9)	0.013
Previous spontaneous bacterial peritonitis (n, %)	24 (10)	6 (8)	0.586
Previous hepato-renal syndrome (n, %)	13 (6)	9 (12)	0.046
HCC within Milan criteria (n, %)	28 (12)	4 (5)	0.107
Active awaiting list for LT (n, %)	37 (16)	4 (5)	0.022
Active alcohol consumption (n, %)	23 (10)	24 (32)	0.001
Etiological treatment (<12 month or during the treatment) (n, %)	70 (30)	44 (59)	0.001
**Concomitant medications**			
Antialdosteronic – daily dosage (mg)	200 (100-200)	200 (200-300)	0.090
Furosemide – daily dosage (mg)	50 (25-75)	50 (25-75)	0.576
Non-Selective beta-blockers (n, %)	118 (50)	34 (45)	0.501
Rifaximin (n, %)	99 (42)	23 (31)	0.086
Fluoroquinolones for SBP prophylaxis (n, %)	7 (3)	4 (5)	0.355
**Comorbidities**			
Chronic Heart Disease (n, %)	44 (19)	10 (13)	0.297
Chronic kidney disease (n, %)	39 (17)	6 (8)	0.075
Chronic lung disease (n, %)	25 (11)	5 (7)	0.320
Neurologic diseases (n, %)	9 (4)	3 (4)	0.937
Diabetes (n, %)	94 (40)	16 (21)	0.004
**Laboratory and hemodynamic data at inclusion**			
Hb (g/dL)	10.6 (9.4-12.2)	10.9 (9.8-12.8)	0.233
WBC (10^9/L)	5.0 (3.8-6.9)	6.4 (4.5-9.0)	0.001
Platelets (10^9/L)	99 (66-137)	120 (80-165)	0.005
Sodium (mmol/L)	136 (133-139)	136 (133-139)	0.701
Bilirubin (mg/dL)	2.1 (1.3-3.7)	2.2 (1.0-4.0)	0.835
Creatinine (mg/dL)	1.0 (0.8-1.3)	0.9 (0.7-1.1)	0.052

Albumin (g/L)	31 (27-35)	31 (27-35)	0.460
INR	1.4 (1.2-1.6)	1.4 (1.2-1.6)	0.591
MAP (mmHg)	85 (78-90)	87 (77-93)	0.474
HR (bpm)	72 (68-81)	77 (70-84)	0.052
**Prognostic scores**			
Child-Pugh score	8 (7-10)	9 (7-10)	0.251
MELD	15 (12-18)	14 (11-18)	0.305
MELD-Na	18 (15-21)	18 (14-21)	0.658

Data is reported by median and interquartile range or absolute frequency and percentage (%) as appropriate. Comparisons between groups were performed by means of Student's t test, Mann–Whitney U test or the chi2 test when appropriate. Abbreviations: MASLD: metabolic dysfunction-associated steatotic liver disease; HCC: hepatocellular carcinoma; LT: liver transplantation; NSBB: nonselective beta blockers; SBP: spontaneous bacterial peritonitis; Hb: haemoglobin; WBC: white blood cells; INR: International Normalized Ratio; MAP: mean arterial pressure; HR: heart rate; MELD: Model for End-stage Liver disease; MELD-Na: Model for End-stage Liver disease incorporating serum sodium.
